# Pericardial application as a new route for implanting stem‐cell cardiospheres to treat myocardial infarction

**DOI:** 10.1113/JP275548

**Published:** 2018-05-07

**Authors:** Jianhua Zhang, Zheng Wu, Zepei Fan, Zixi Qin, Yingwei Wang, Jiayuan Chen, Maoxiong Wu, Yangxin Chen, Changhao Wu, Jingfeng Wang

**Affiliations:** ^1^ Department of Cardiology The Sun Yat‐sen Memorial Hospital of Sun Yat‐sen University Guangzhou 510120 PR China; ^2^ Department of Cardiology The First Affiliated Hospital of Jinan University Guangzhou PR China; ^3^ Key Laboratory for Regenerative Medicine, Ministry of Education Jinan University Guangzhou PR China; ^4^ Faculty of Health and Medical Sciences, School of Biosciences and Medicine University of Surrey Guildford GU2 7XH U.K.

**Keywords:** cardiospheres, pericardial fluid from rats with myocardial infarction, matrix hydrogel, pericardial cavity, myocardial infarction

## Abstract

**Key points:**

- Cardiospheres (CSps) are a promising new form of cardiac stem cells with advantage over other stem cells for myocardial regeneration, but direct implantation of CSps by conventional routes has been limited due to potential embolism.
- We have implanted CSps into the pericardial cavity and systematically demonstrated its efficacy regarding myocardial infarction.
- Stem cell potency and cell viability can be optimized *in vitro* prior to implantation by pre‐conditioning CSps with pericardial fluid and hydrogel packing.
- Transplantation of optimized CSps into the pericardial cavity improved cardiac function and alleviated myocardial fibrosis, increased myocardial cell survival and promoted angiogenesis.
- Mechanistically, CSps are able to directly differentiate into cardiomyocytes *in vivo* and promote regeneration of myocardial cells and blood vessels through a paracrine effect with released growth factors as potential paracrine mediators.
- These findings establish a new strategy for therapeutic myocardial regeneration to treat myocardial infarction.

**Abstract:**

Cardiospheres (CSps) are a new form of cardiac stem cells with an advantage over other stem cells for myocardial regeneration. However, direct implantation of CSps by conventional routes to treat myocardial infarction has been limited due to potential embolism. We have implanted CSps into the pericardial cavity and systematically assessed its efficacy on myocardial infarction. Preconditioning with pericardial fluid enhanced the activity of CSps and matrix hydrogel prolonged their viability. This shows that pretransplant optimization of stem cell potency and maintenance of cell viability can be achieved with CSps. Transplantation of optimized CSps into the pericardial cavity improved cardiac function and alleviated myocardial fibrosis in the non‐infarcted area, and increased myocardial cell survival and promoted angiogenesis in the infarcted area. Mechanistically, CSps were able to directly differentiate into cardiomyocytes *in vivo* and promoted regeneration of myocardial cells and blood vessels in the infarcted area through a paracrine effect with released growth factors in pericardial cavity serving as possible paracrine mediators. This is the first demonstration of direct pericardial administration of pre‐optimized CSps, and its effectiveness on myocardial infarction by functional and morphological outcomes with distinct mechanisms. These findings establish a new strategy for therapeutic myocardial regeneration to treat myocardial infarction.

## Key points

- Cardiospheres (CSps) are a promising new form of cardiac stem cells with advantage over other stem cells for myocardial regeneration, but direct implantation of CSps by conventional routes has been limited due to potential embolism.
- We have implanted CSps into the pericardial cavity and systematically demonstrated its efficacy regarding myocardial infarction.
- Stem cell potency and cell viability can be optimized *in vitro* prior to implantation by pre‐conditioning CSps with pericardial fluid and hydrogel packing.
- Transplantation of optimized CSps into the pericardial cavity improved cardiac function and alleviated myocardial fibrosis, increased myocardial cell survival and promoted angiogenesis.
- Mechanistically, CSps are able to directly differentiate into cardiomyocytes *in vivo* and promote regeneration of myocardial cells and blood vessels through a paracrine effect with released growth factors as potential paracrine mediators.
- These findings establish a new strategy for therapeutic myocardial regeneration to treat myocardial infarction.

## Introduction

Ischaemic heart disease associated with myocardial infarction is the leading cause of mortality (Mozaffarian *et al*. 2015). Although revascularization therapy can improve the survival of patients with myocardial infarction, it cannot resolve ventricular remodelling and heart failure as a result of myocardial damage (St John Sutton *et al*. 2003; Daubert *et al*. 2015). How to regenerate myocardial cells and make up the loss caused by myocardial infarction is a pressing medical issue. Experimental and clinical studies show that stem cell therapy can improve cardiac function and prognosis (Fisher *et al*. 2015).

Many sources of stem cells have been used in experimental models and clinical trials of myocardial infarction, including bone marrow‐derived stem cells or skeletal myoblast‐derived stem cells, and more recently stem cells derived from the heart. While bone marrow‐derived cells do not engraft well over the long term, skeletal myoblast‐derived stem cells are pro‐arrhythmic. Cardiac stem cells may offer advantage over extracardiac stem cells in differentiating into cardiac myocytes and better survival in the myocardium (Messina *et al*. 2004; Bearzi *et al*. 2007). How to apply these cells effectively in myocardial regeneration is of intense interest.

Cardiospheres (CSps) are a particular form of stem cells derived *in vitro* from *in situ* stem cells of cardiac tissue. The structure of CSps mimics the niche microenvironment of cardiac stem cells *in vivo* with undifferentiated cardiac stem cells in the core and cardiac‐committed cells on the outer layer (Chimenti *et al*. 2010). This gradient structure is beneficial to the survival of stem cells and maintenance of their ‘stemness’. Intact, stem cell‐containing CSps are different from isolated stem cells derived from the CSps (cardiosphere‐derived cells, CDCs) (Barile *et al*. 2013) and produce stronger paracrine effects than the latter, and have the ability to directly differentiate into myocardial cells (Davis *et al*. 2010; Barile *et al*. 2013), and thus have greater potential for myocardium regeneration. However, they are small particles in nature. This limits the pathways for transplantation *in vivo* due to potential embolism. The conventional delivery routes are not well suited to implantation of CSps and are associated with very low survival rates in the heart tissue (Hou *et al*. 2005). The pericardial cavity is a double‐walled sac, which would prevent stem cells and spheres from entering the blood circulation, but proteins with a molecular mass below 40 kDa can diffuse through the epicardium into the pericardial space (Limana *et al*. 2010), which allows paracrine communications. We hypothesize that intrapericardial delivery is an effective way for direct CSps transplantation, which allows safe implantation, avoids the risk of entry into the bloodstream and produces a regenerative effect. We reason that pericardial cavity also allows the transplanted stem cells to be preconditioned or pre‐treated in order to generate better therapeutic effects after transplantation. Our second hypothesis is that the activity of CSps can be further improved by pericardial fluid (PF) obtained from another subject preconditioned with myocardial infarction, as PF from human with myocardial infarction, when injected into the pericardial cavity in mice, can increase the expression of stem cell genes, especially c‐kit, in epicardium (Limana *et al*. 2010). Therefore, the present study aimed first to enhance the efficacy of CSps by pretreatment with PF from rats with myocardial infarction (PFMI) and secondly to assess the therapeutic effect on myocardial infarction by administering these functionally enhanced CSps into the pericardial cavity. One challenge in cell transplantation is how to maintain the activity of stem cells during transfer from the laboratory to the operating theatre (Hanna & Hubel, 2009; Schoenhard & Hatzopoulos, 2010). A further objective was therefore to improve the survival of CSps *in vitro* before transplantation, by packaging CSps with matrix hydrogel before *in vivo* application.

We therefore first evaluated the effects of different concentrations of PFMI on CSps *in vitro*, and selected the most efficacious CSps as the target stem cells. We then assessed the protective effect of matrix hydrogel on the survival of CSps *in vitro*. The selected most potent CSps packaged by matrix hydrogel were then transplanted into the pericardial cavity in rats undergoing myocardial infarction. The survival and outcome of CSps, the effect of its transplantation on myocardial infarction and the possible mechanisms were examined.

## Methods

### CSps culture

Specimen preparation, CSps culturing and harvesting were performed according to a previously published method (Stastna *et al*. 2010), but with a different culture medium for CSps. Briefly, cardiac tissue specimens from the septum of the left ventricle of SD rats (male, 3 weeks old) were cut into small pieces, washed in PBS three times, partially enzymatically digested and grown in primary cultures as explants on poly‐d‐lysine (Sigma, St. Louis, MO, USA) coated dishes in complete explant medium (CEM). The CEM medium consisted of Iscove's modified Dulbecco's medium (Corning, Corelle, NY, USA) supplemented with 10% FBS (Corning), 1% penicillin‐streptomycin, 1% l‐glutamine and 0.1 mmol l^−1^ 2‐mercaptoethanol, at 37°C and 5% CO_2_. The culture medium was replaced every 2–3 days. After 1–2 weeks, round phase bright cells, migrating over a layer of fibroblast‐like cells derived from explants, were harvested by mild trypsinization and seeded at about 7 × 10^4^ cells cm^−2^ onto poly‐d‐lysine coated wells in CEM. About 1 week after plating, CSps of passage 0 (P0) were collected for passage. The number of CSps per generation was recorded.

### Immunofluorescence staining

Cells were incubated with the primary antibodies at 4°C overnight. These primary antibodies were anti‐c‐kit (ab8295), anti‐sca‐1 (ab4336) and anti‐KDR (ab9530) (Abcam, Cambridge, MA, USA). After being washed three times with PBST, cells were incubated with Alexa Fluor 488 goat anti‐mouse (A11001, Invitrogen, Carlsbad, CA, USA) or Alexa Fluor 594 goat anti‐rabbit (AB6939, Abcam) antibodies in 1% BSA for 1 h at room temperature in the dark. A laser scanning confocal microscope (Nikon Eclipse Ti, Nikon, Tokyo, Japan) and the supplier software were used for imaging and analysis.

### Effect of PFMI on CSps

The PF was aspirated under sterile conditions from 10 rats that were euthanized 4 days after myocardial infarction, centrifuged for 10 min at 3000 *g* at 4°C, mixed together and passed through a 0.22 μm filter to remove cell debris. Cell suspensions of CSps were passaged at a density of 5000 cells cm^−2^ in 96‐well plates, and CSps were formed again after 3 days. Different concentrations of PF were added (0, 25, 50 and 100%), and after 24 h of culture, CSps were made into single cell suspensions and seeded to the plates in culture medium (without phenol‐red). Cell activity was detected according to the CCK‐8 (Sigma) operation manual and absorbance was read at 450 nm. CSps were collected, and gene expression levels of VEGF, bFGF, FGF, IGF‐1, cTnT, c‐kit, sca‐1 and KDR were detected by quantitative RT‐PCR (qRT‐PCR).

### Quantitative RT‐PCR

mRNA levels of VEGF, bFGF, HGF and IGF‐1were determined by qRT‐PCR. In brief, total RNA was extracted from cultured CSps using Trizol reagent (Invitrogen) as per the manufacturer's instructions. RNA was reverse transcribed to cDNA using a PrimeScriptTM RT reagent kit (TaKaRa, Dalian, China). Reverse transcription was performed at 37°C for 15 min and 85°C for 5 s. Real‐time PCR amplification was performed using a LightCycler 480 Real‐Time PCR System (Roche, Switzerland). After amplification, a melting curve was acquired by heating at 4°C s^–1^ to 95°C, cooling at 4°C s^–1^ to 70°C, maintenance at 70°C for 20 s, and then slowly heating at 4°C s^–1^ to 95°C to determine the specificity of PCR products. All qRT‐PCR data were normalized to the reference gene GAPDH. The PCR primer sequences were as follows: VEGF, 5’‐CGACAGAAGGGGAGCAGAAA‐3’ (forward primer) and 5’‐GCTGGCTTTGGTGAGGTTTG‐3’(reverse primer); bFGF, 5’‐GATCCCAAGCGGCTCTACTG‐3’ (forward primer) and 5’‐CCGTGACCGGTAAGTGTTGT‐3’(reverse primer); HGF, 5’‐CCTTCGAGCTATCGCGGTAA‐3’ (forward primer) and 5’‐GAATTTGTGCCGGTGTGGTG‐3’(reverse primer); IGF‐1, 5’‐CAAAATGAGCGCACCTCCAA‐3’ (forward primer) and 5’‐CTTCAGCGGAGCACAGTACA‐3’(reverse primer); GAPDH, 5’‐AAGGTCGGAGTCAACGGATTT‐3’ (forward primer) and 5’‐AGATGATGACCCTTTTGGCTC‐3’(reverse primer); c‐kit, 5’‐AATCCGACAACCAAAGCAAC‐3’ (forward primer) and 5’‐ACCACAGGTTGAGACTACAGT‐3’(reverse primer); sca‐1, 5’‐AACCATATTTGCCTTCCCGTCT‐3’ (forward primer) and 5’‐CCAGGTGCTGCCTCCAGTG‐3’(reverse primer); KDR, 5’‐ATTCTGGACTCTCCCTGCCTA‐3’ (forward primer) and 5’‐TGTCTGTCTTGGCTGTCATCTG‐3’(reverse primer); c‐TnT, 5’‐AGAGGACTCCAAACCCAAGC‐3’ (forward primer) and 5’‐ATTGCGAATACGCTGCTGTT‐3’(reverse primer).

### DiR label and preparation of matrix hydrogel CSps suspension

CSps were labelled with 3.5 μg mL^−1^ of 1,1′‐dioctadecyl‐3,3,3′‐tetramethylindotricarbocyanine iodide (DiR, Caliper Life Sciences, Waltham, MA, USA) by addition of the dye into cells suspended in PBS (Granot *et al*. 2007). After 30 min of incubation at 37°C, cells were extensively washed in PBS. About 5 × 10^6^ cells were collected in the centrifuge tube. Then, 100 μl matrix hydrogel (1:2 dilution) (Corning) was added to the tube and mixed thoroughly.

### Flow cytometry analysis

Cell apoptosis and necrosis were assessed using an Annexin V‐FITC/PI apoptosis kit (Life Technologies Inc., Carlsbad, CA, USA) as per the manufacturer's instructions. Cells were divided randomly into six groups: CSps were collected in PBS at three time points (PBS 1 h, PBS 3 h, PBS 6 h) or in Matrix hydrogel at the same time points (Matrigel 1 h, Matrigel 3 h, Matrigel 6 h). At each time point, CSps were washed in PBS and dissociated to generate single cell suspensions. The cells were incubated with 5 μL Annexin V and 5 μL PI (Becton Dickinson, Franklin Lakes, NJ, USA) for 10 min at room temperature in the dark. Cells from each sample were then analysed using a FacsCalibur flow cytometer (Becton Dickinson). The data were analysed using CELLQuest software (Becton Dickinson). The results were interpreted as follows: cells in the lower‐left quadrant (Annexin‐V^−^/PI^−^) represent living cells; those in the lower‐right quadrant (Annexin‐V^+^/PI^−^) represent early apoptotic cells; those in the upper‐right quadrant (Annexin‐V^+^/PI^+^) represent late apoptotic cells; and those in the upper‐left quadrant (Annexin‐V^−^/PI^+^) represent necrotic cells. Experiments were repeated at least three times independently.

### Experimental protocol and animal surgery

All animal handling and procedures were performed in accordance with the protocols for these studies approved by Animal Ethics Committee of Sun Yat‐sen University. All animals received humane care in compliance with the Guide for the Care and Use of Laboratory Animals (National Institutes of Health, revised 1996). Surgery was performed under deep anaesthesia and every attempt was made to minimize pain and discomfort. Left coronary artery occlusion was performed in SD rats (female, 250–280 g) as previously described (Zhang *et al*. 2010). Briefly, after anaesthesia by intraperitoneal administration of pentobarbital sodium (30 mg kg^−1^, Sigma), all animals underwent endotracheal intubation. Mechanical ventilation was provided with room air at 60 to 70 breaths min^–1^ using a Rodent Respirator (Taimeng Company, Chengdu, China). A standard lead‐II ECG was recorded via subcutaneous stainless‐steel electrodes. After a left thoracotomy was performed to expose the heart at the fifth intercostal space, the left anterior descending coronary artery was ligated with a 6‐0 silk suture. Ischaemia was confirmed by the elevation of ST segment in ECG and cardiac cyanosis. After these surgical procedures, rats were allowed to stabilize for 15 min. Matrigel CSps suspensions (0.1 mL) or blank Matrigel were injected in the pericardial cavity through the trocar (24 G, B. Braun Melsungen AG, Melsungen, Germany). The pericardial membrane was then sutured to close the pericardial cavity. The sham‐operated rats underwent the same operative procedures, but the suture was loosely tied to avoid coronary artery occlusion. The rats undergoing myocardial infarction operation were divided into three groups: CSps group (*n* = 25), Control group (*n* = 25) and Sham group (*n* = 25). Rats were given Matrigel CSps suspensions (5 × 10^6^ cells) for the CSps group, and blank Matrigel only for the Control and Sham groups.

### Small animal *in vivo* imaging technology

Xenogen's IVIS 100 Series Imaging System (Alameda, CA, USA) and Olympus SZX12 (Tokyo, Japan) microscope, coupled with a Pixelfly QE (PCO, Kelheim, Germany) charge‐coupled device (CCD) camera, were used to monitor localization of DiR‐labelled CSps within live animals (Kalchenko *et al*. 2006). Imaging was performed 1, 2 and 4 weeks after CSps injection, and the survival rate of CSps was calculated by dividing the fluorescence value of DiR at each time point with that immediately after injection. The excitation and emission filters in the IVIS system were set at 760 and 810 nm, respectively. Image processing and data analysis were performed using Living Image 2.5 software and ImageJ version 1.34 (W. Rasband, National Institutes of Health, Bethesday, MD, USA). The experiments were repeated in at least six different animals per group.

### Functional evaluation of the left ventricle

Transthoracic echocardiography (ultrasound cardiography, UCG) was performed with animals anaesthetized. Two‐dimensional short‐ and long‐axis images of the left ventricle were obtained at the papillary muscle level (ULTRAMARK 9, Philips, USA). The following parameters were measured: left ventricular anterior wall thickness (AWT), left ventricle end‐systolic dimension (LVESD) and left ventricle ejection fraction (LVEF). Four different time points were selected for echocardiographic studies: before (before the surgical procedure), and 1, 2 and 4 weeks after myocardial infarction.

### Small‐animal PET/CT scanning

At 4 weeks after myocardial infarction, positron emission tomography (PET) and image analysis were performed using an Inveon micsoPET scanner (Siemens Medical Solutions, Erlangen, Germany) (Gao *et al*. 2012). Animals were anaesthetized as described above, and approximately 100 μCi 100 g^−1^ body weight of 2‐deoxy‐2‐[fluorine‐18]fluoro‐D‐glucose (F‐FDG, gift from the First Affiliated Hospital of Sun Yat‐sen University) was administered via tail vein injection. For each scan, regions of interest (ROIs) were drawn using the software on decay‐corrected whole‐body coronal images. Radioactivity levels within the myocardium were obtained from mean pixel values within the multiple ROI volumes and then converted to MBq mL^–1^. These values were then divided by the administered activity to obtain (assuming a tissue density of 1 g mL^−1^) an image ROI‐derived percentage injected dose per gram (%ID g^–1^). The ability to uptake F‐FDG in the infarct zone represents the number of viable myocardial cells.

### Tissue processing and staining

For histological analysis, six animals per group were sacrificed (via with an overdose of pentobarbital sodium) at 4 weeks after myocardial infarction and CSps transplantation. Hearts were quickly removed and fixed with 4% paraformaldehyde for Masson tri‐chrome stain and immunohistochemistry.

The components of cardiac tissue were identified according to their colour by Masson stain: blue for collagen fibres and red for myocytes. Myocardial infarct size was measured as the percentage of the left ventricle circumference occupied by scar tissue in the mid‐left ventricle tissue section slices. Collagen volume fraction (CVF) was determined using an HPISA 100 chromatic colour pathological analysis system (Olympus) using five random images from each slide and five slides per sample, and the mean values of CVF were obtained by one investigator blinded to the experimental groups.

For immunohistochemistry, transverse myocardial sections (4 μm thick) were deparaffinized and treated with 0.3% hydrogen peroxide and then blocked with 10% normal serum (homologous with the primary antibodies) for 30 min at room temperature. The following primary antibodies were used: cTnT (ab8295, Abcam) and a‐SMA (ab7817, Abcam). The ROI of cTnT was used to evaluate myocardial cells in the infarcted region. Arteriolar density (arterioles per field) was quantified in a similar way after staining with α‐SMA in separate sections. In each rat heart, 3 sections were chosen and in every section three fields were randomly selected for computerized planimetry. After staining, the average positive area was calculated by computer‐assisted planimetry (Image‐Pro Plus 6.0, Media Cybernetics, Silver Springs, MD, USA). 5‐Bromo‐2‐deoxyuridine (BrdU, Sigma, B5002) was used to detect the proliferation of new cells *in vivo* after myocardial infarction. Each group of rats was given BrdU (i.p. injection, 100 mg kg day^−1^) for 4 weeks after myocardial infarction. Paraffin sections of cardiac tissue were de‐waxed and rehydrated, and the antigen was retrieved. The slices were then incubated in diluted hydrochloric acid for 30 min to denature DNA. Thereafter, the tissue sections were blocked using 10% goat serum for 1 h, incubated with primary BrdU antibody (Abcam, ab8039) in 1:1000 dilution overnight at 4°C, followed by incubation with secondary antibody (Invitrogen, A10521) for 2 h at room temperature. The nucleus was labelled by Hoechst 33258.

### ELISA

Pericardial fluid was directly collected 1 week after myocardial infarction, and pericardial lavage fluid was collected after 4 weeks. Four weeks after myocardial infarction, animals were anaesthetized and underwent endotracheal intubation. Mechanical ventilation was provided using a Rodent Respirator in order to keep the heart beating. Then, 100 μl sterile double distilled water was injected into the pericardial cavity, and the fluid was collected after being mixed by the heart beating for 10 min. The levels of VEGF, bFGF, HGF and IGF‐1 in PF or pericardial lavage fluid were measured by an enzyme‐linked immunosorbent assay (ELISA; R&D, Minneapolis, MN, USA), according to the instructions supplied.

## Statistical analysis

All data are expressed as mean ± SD. Statistical analysis was performed with SPSS 13.0. One‐way ANOVA was used to evaluate differences among three groups or a *t* test between two groups. Two‐way ANOVA was used to analyse the difference among three groups at multiple time points. A value of *P* < 0.05 was considered statistically significant.

## Results

### Characteristics of CSps *in vitro*

**Figure 1:**
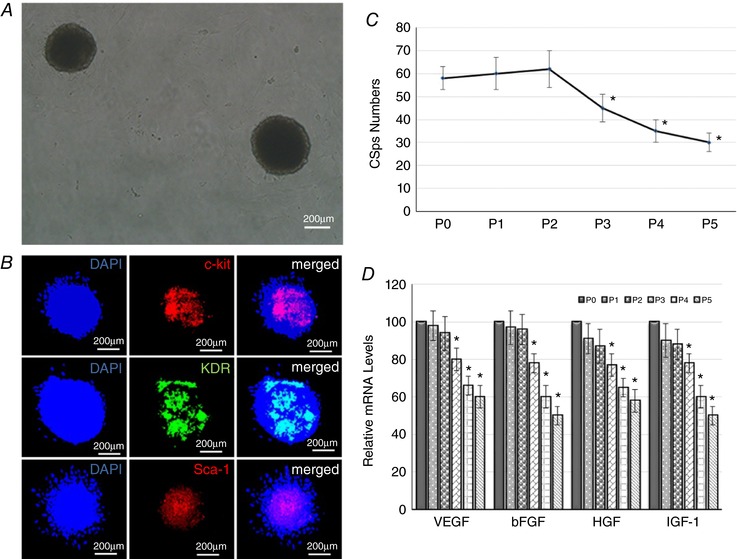
Characteristics of CSps in culture *A*, morphology of CSps in *in vitro* culture (P2 generation); *B*, number of CSps generated in different passages (*n* = 10 dishes per generation); *C*, expression of c‐kit, sca‐1 and KDR in CSps by immunofluorescence staining (*n* = 5); *D*, gene expression levels of VEGF, bFGF, HGF and IGF‐1 in different culture passages by real‐time quantitative PCR (*n* = 3 per generation). ^*^ *P* < 0.05 *versus* P0.

The characteristics of CSps *in vitro* are shown in Fig. 1. In Fig. 1 *A*, CSps were formed as three‐dimensional spheres in the culture *in vitro*. Expression of the cardiac stem cells markers is shown in Fig. 1 *B*. It can be seen that c‐kit, sca‐1 and KDR were all expressed in the interior of CSps, which simulates the niche structure of cardiac stem cells in the growth microenvironment *in vivo*. However, the locations of the three markers in the CSps were different; c‐kit and KDR were not fixed, but sca‐1 was always expressed in the centre of CSps. We also noticed that the cells expressing any one of the three markers, c‐kit, sca‐1 and KDR, would certainly form CSps, and none of the three markers was expressed in the monolayer cells (data not shown). Figure 1 *C* shows the difference in the amount of CSps generated in different culture passages. There was no significant difference in the number of clones between P1 (56 ± 7), P2 (55 ± 8) and P0 (58 ± 5). However, from P3 (45 ± 6), the number of clones gradually decreased (35 ± 5 for P4 and 56 ± 7 for P5), and the difference was statistically significant (*P* < 0.05). In the present study, passages P0–P5 of CSps were collected to measure the gene expression level of the growth factors by qRT‐PCR. Figure 1 *D* shows the expression levels of growth factors VEGF, bFGF, HGF and IGF‐1, in different passages of CSps. From P3, the expression of VEGF (80 ± 6%), bFGF (78 ± 5%), HGF (77 ± 6%) and IGF‐1 (78 ± 6%) was significantly reduced, but there was no significant difference among P1, P2 and P0. According to the above results, the P2 generation of CSps was selected as the candidate stem cells for transplantation *in vivo*.

### Protective effect of matrix hydrogel on CSps *in vitro*

**Figure 2:**
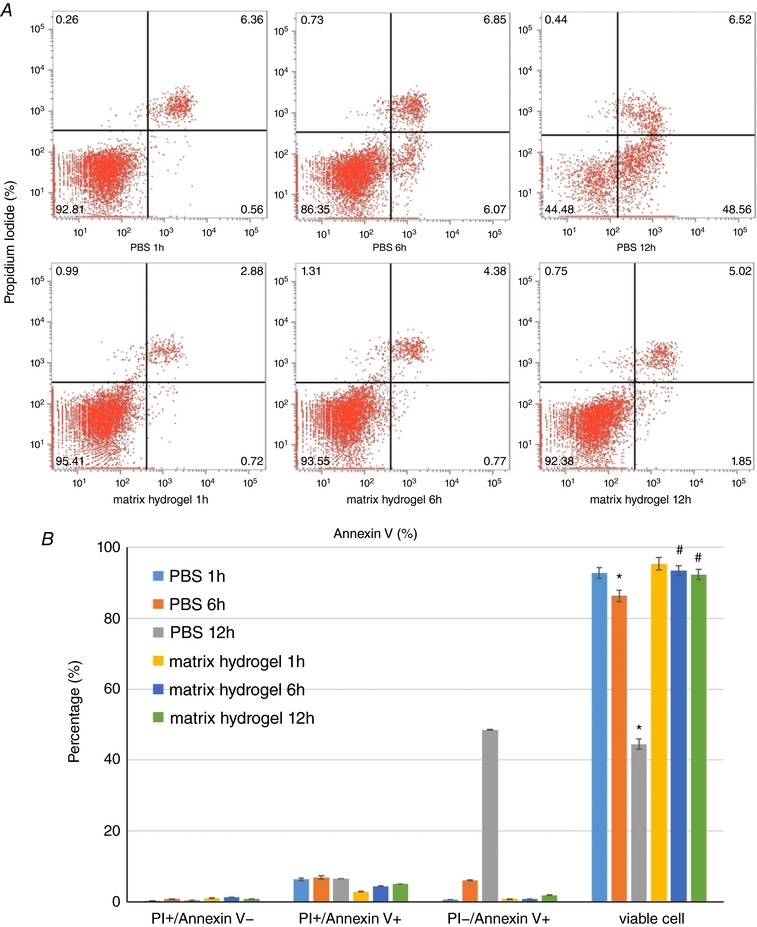
Protective effect of matrix hydrogel on CSps *A*, survival, apoptosis or death of CSps detected by flow cytometry; *B*, averaged data of survival, apoptosis or death of CSps and statistical analysis. ^*^ *P* < 0.05 *versus* PBS 1 h group; ^#^ *P* < 0.05 *versus* PBS groups at the same time points.

The protective effect of matrix hydrogel on CSps *in vitro* is shown in Fig. 2. At 4°C, CSps in the PBS control group began to be apoptotic after 1 h *in vitro*, and the proportion of living cells continued to decrease with time. After 1, 6 and 12 h, the proportion of living cells in good condition was only 92.81 ± 1.48, 86.35 ± 1.65 and 44.48 ± 1.45%, respectively. Compared with the PBS control group, the survival rate of CSps in the matrix hydrogel was significantly increased. The proportions of living CSps were 95.41 ± 1.83, 93.55 ± 1.36 and 92.38 ± 1.43%, respectively, after 1, 6 and 12 h *in vitro* at 4°C.

### Effect of PFMI on the biological characteristics of CSps

**Figure 3:**
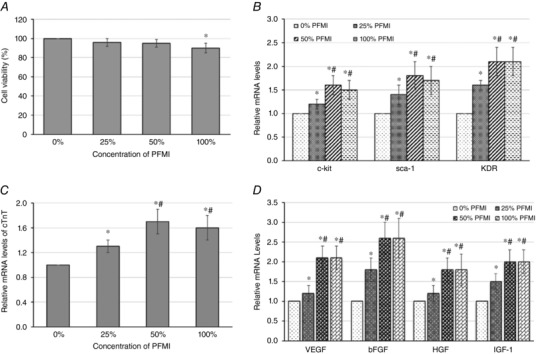
Effect of different concentrations of PFMI pretreatment on biological characteristics of CSps *A*, effect on cell viability; *B*, effect on mRNA expression of c‐kit, sca‐1 and KDR; *C*, effect on mRNA expression of cTnT; *D*, effect on mRNA expression of VEGF, bFGF, HGF and IGF‐1. ^*^ *P* < 0.05 *versus* 0% PFMI; ^#^ *P* < 0.05 *versus* 25% PFMI. PFMI, pericardial fluid from rats with myocardial infarction.

Figure 3 shows the effect of different concentrations of PFMI on the biological characteristics of CSps. Figure 3 *A* shows the effect on the viability of CSps. There was no significant effect on cell viability after pretreatment with 25 and 50% PFMI, although the cell viability of CSps was slightly decreased after pretreatment with 100% PFMI (*P* < 0.05). The results from Fig. 3 *B* show that all concentrations of PFMI pretreatment were able to enhance the gene expression of c‐kit, sca‐1 and KDR. The effect was significantly greater with 50 and 100% PFMI pretreatment (*P* < 0.05, *vs*. 25% PFMI); however, there was no significant difference between 50 and 100% PFMI pretreatment. As shown in Fig. 3 *C*, pretreatment of PFMI promoted cTnT mRNA expression, with a greater effect for 50 and 100% PFMI (*P* < 0.05, *vs*. 25% PFMI). Figure 3 *D* shows that PFMI pretreatment promoted the mRNA expression of VEGF, bFGF, HGF and IGF‐1 in CSps. Compared with 25% PFMI, 50 and 100% PFMI produced a stronger effect (*P* < 0.05). All the data in Fig. 3 demonstrate that PFMI at all concentrations generally enhanced the expression of major stem cell genes and cardiac muscle‐specific genes of CSps, and also the expression of growth factors, hence indicating a higher biological activity of CSps with minimal adverse effects on cell viability. In particular, 50% PFMI pretreatment was able to significantly enhance the biological activity of CSps, but had no significant effect on cell viability. This concentration of CSps was therefore used for *in vivo* experiments.

### Survival and outcome of CSps after transplantation *in vivo*

**Figure 4:**
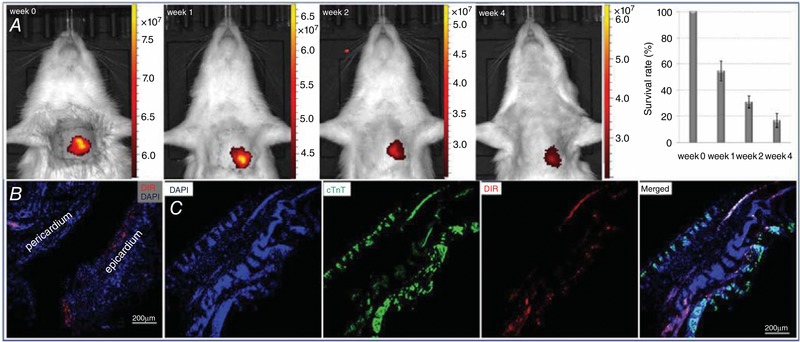
Survival and the outcome of CSps after transplantation *in vivo* *A*, survival rate of DiR‐labelled CSps *in vivo* at week 1, 2 and 4; *B*, DiR‐labelled CSps survived and infiltrated to the epicardium at week 4; *C*, DiR‐labelled CSps migrated into infarcted myocardial tissue at week 4. Immunofluorescence data: DiR = 1,1′‐dioctadecyl‐3,3,3′‐tetramethylindotricarbocyanine iodide, DAPI = 4′,6‐diamidino‐2‐phenylindole, cTnT = cardiac troponin T. Note, DAPI and cTnT images show loss of myocardial cells in the infarcted myocardial tissue.

The *in vivo* survival rate of DiR‐labelled CSps at different time points was detected using a small animal *in vivo* imaging technique. The results are shown in Fig. 4 *A*. The survival rates of CSps *in vivo* were 54.5 ± 7.5% at week 1, 31.0 ± 4.5% at week 2 and 16.8 ± 5.3% at week 4. Furthermore, all DiR‐labelled CSps were distributed inside cardiac tissue, and no fluorescence was seen in the left or right lung.

The fate of CSps in cardiac tissue after transplantation is shown in Fig. 4 *B* and *C*. At 4 weeks after transplantation, DiR‐labelled cells were clearly seen in cardiac tissue sections, suggesting survival in cardiac tissue *in vivo*. Specifically, these cells reached the epicardium (Fig. 4 *B*), and also migrated into the infarcted myocardial tissues (Fig. 4 *C*). These data show that the implanted CSps were maintained well in the heart with no loss to other tissues and were able to migrate to myocardial tissue and infiltrate into the infarcted area.

### LV function and survival rate of rats after myocardial infarction

**Figure 5:**
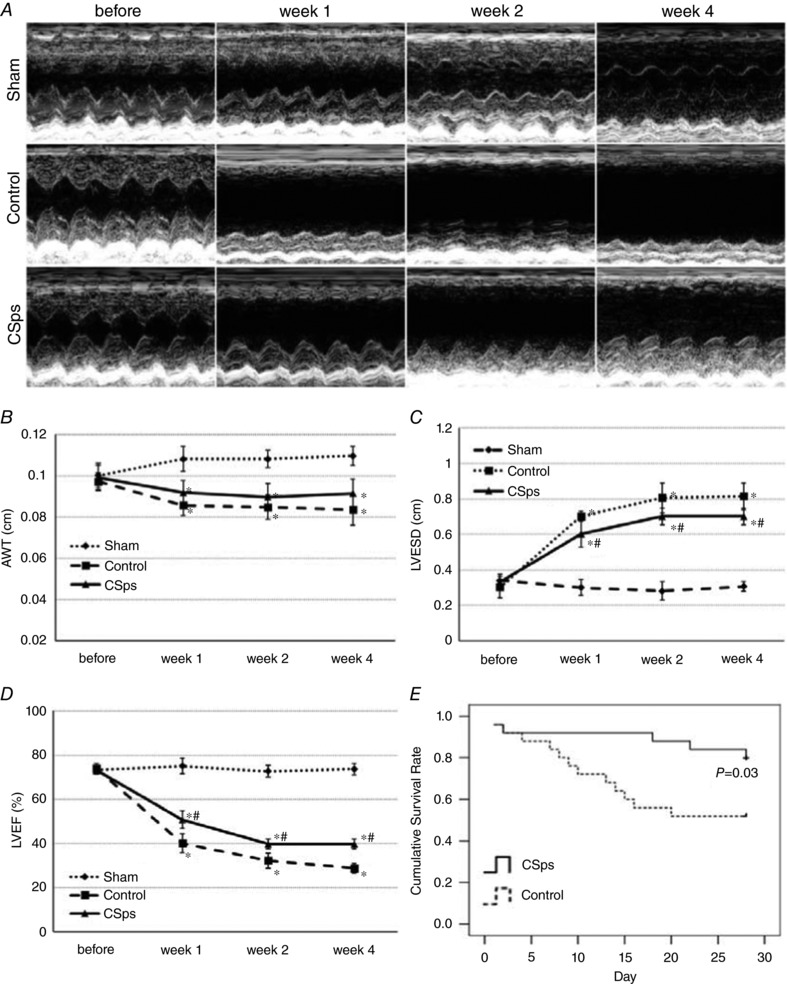
Effect of CSps administration on cardiac function and the survival of rats undergoing myocardial infarction *A*, M‐mode ultrasound at level of short axis of left ventricle; *B*, results of AWT; *C*, results of LVESD; *D*, results of LVEF. Two‐way ANOVA results of data in *B*, *C* and *D* are given in Table 2; *E*, Kaplan–Meier curves for the survival of rats in the CSps (*n* = 25) and Control groups (*n* = 25). ^*^ *P* < 0.05 *versus* Sham group; ^#^ *P* < 0.05 *versus* Control group.

The UCG results before and after myocardial infarction are shown in Fig. 5 and Table 2. Figure 5 *A* and *B* shows that after myocardial infarction, AWT was smaller in the CSps and Control groups, compared with the Sham group (*P* < 0.05). The difference in AWT between the CSps and Control groups was not statistically significant, despite higher absolute values at different time points (Fig. 5 *B*). Myocardial infarction caused an increase in LVESD as seen in infarction Control. However, CSps transplantation significantly inhibited the increase in LVESD caused by myocardial infarction with an approximately 15% reduction, an effect which occurred with an early onset and was maintained throughout the experiment (*P* < 0.05 *vs*. Control group, Fig. 5 *A*–*C*). Of note, myocardial infarction reduced LVEF but CSps transplantation significantly increased LVEF by about 40%, an effect seen at all the time points (Fig. 5 *A*–*D*). Figure 5 *E* shows the number of surviving rats in the Sham, CSps and Control groups. The survival rate was reduced to 52% 4 weeks after myocardial infarction (Control). CSps administration increased the survival rate to 80% (*P* < 0.05 *vs*. Control). Kaplan–Meier curves for the survival of rats in the CSps and Control groups are shown in Fig. 5 *E* (*n* = 25 for each group).

### Fibrosis in non‐infarcted area, viable myocardial cells and angiogenesis in the infarcted area

**Figure 6:**
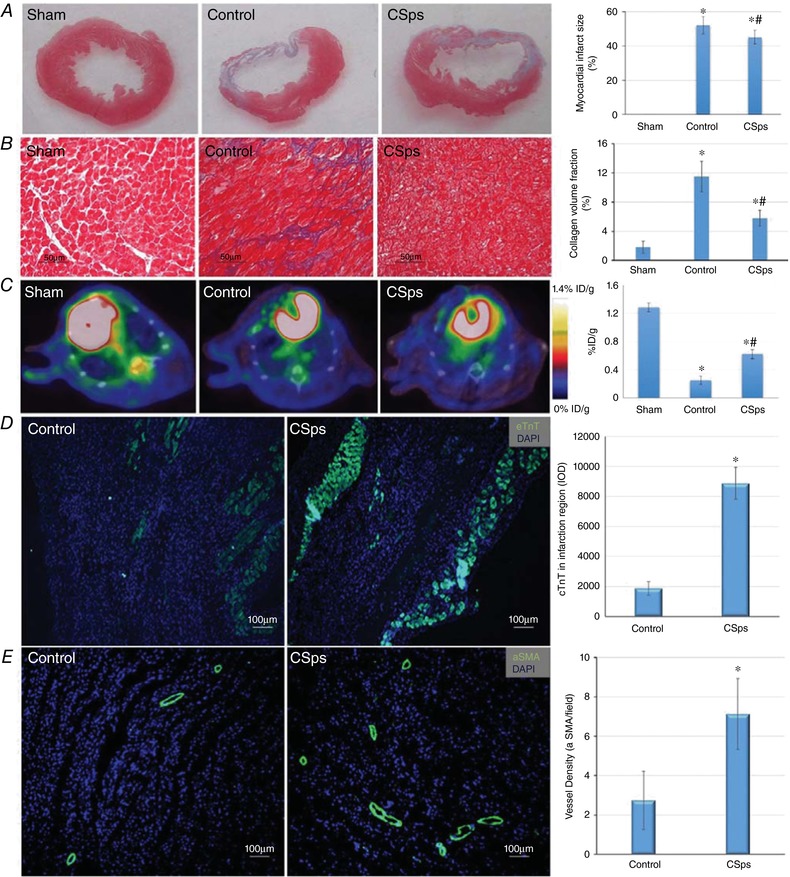
Effect of CSps on collagen volume fraction in non‐infarcted area, viable myocardial cells and angiogenesis in the infarcted area *A*, Masson staining results 4 weeks after myocardial infarction; *B*, effect on collagen volume fraction in non‐infarcted area 4 weeks after myocardial infarction; *C*, F‐FDG PET of Sham, Control and CSps rats at 4 weeks after myocardial infarction; *D*, expression of cTnT in the infarcted myocardial tissue by immunofluorescence; *E*, expression of α‐SMA in the infarcted myocardial tissue by immunofluorescence. ^*^ *P* < 0.05 *versus* Sham group; ^#^ *P* < 0.05 *versus* Control group.

Figure 6 *A* shows that CSps significantly reduced the infarcted size (*P* < 0.05). The deposition of collagen in the non‐infarcted area is shown in Fig. 6 *B*. Masson staining revealed that myocardial infarction resulted in myocardial fibrosis in the non‐infarcted area. CVF was increased significantly in the Control group (11.5 ± 2.1 %) compared with the Sham group (1.8 ± 0.8 %) 4 weeks after myocardial infarction. CSps transplantation significantly inhibited the increase in CVF in the non‐infarcted area with a 50% reduction (5.8 ± 1.1%, *P* < 0.05).

Myocardial cell viability in the infarcted area 4 weeks after myocardial infarction is shown in Fig. 6 *C*. Four weeks after myocardial infarction, there was an apparent uptake defect in the anterolateral wall of the left ventricle in Control rats (as shown by Gao *et al*. 2012) with F‐FDG under micro‐PET/CT (Fig. 6 *C*). However, the uptake rate of F‐FDG was 2.5‐fold higher in the CSps group (0.622 ± 0.064%ID g^–1^ *vs*. 0.246 ± 0.059 %ID g^–1^ in the Control group, *P* < 0.05). This suggests that there were significantly more living cells following CSps administration. Further immunofluorescence determined the expression of myocardial cells (cTnT‐positive) and blood vessels (α‐SMA‐positive) 4 weeks after myocardial infarction. Figure 6 *D* shows that compared with the Control group, CSps transplantation significantly increased the expression of cTnT in the infarcted area, and quantitative image analysis shows that the Integrated Optical Density (IOD) of cTnT was 8884.5 ± 1063.8 in the CSps group compared with 1872 ± 463.5 in the Control group (*P* < 0.05), demonstrating a large effect on myocardial cell regeneration. The effect of transplantation of CSps on angiogenesis in the infarct area is shown in Fig. 6 *E*. The vascular density was also significantly higher in the CSps group (7 ± 2 per field) than in the Control group (3 ± 1 per field) (*P* < 0.05).

### Mechanisms of action – direct differentiation of CSps into cardiac myocytes and the paracrine effect

**Figure 7:**
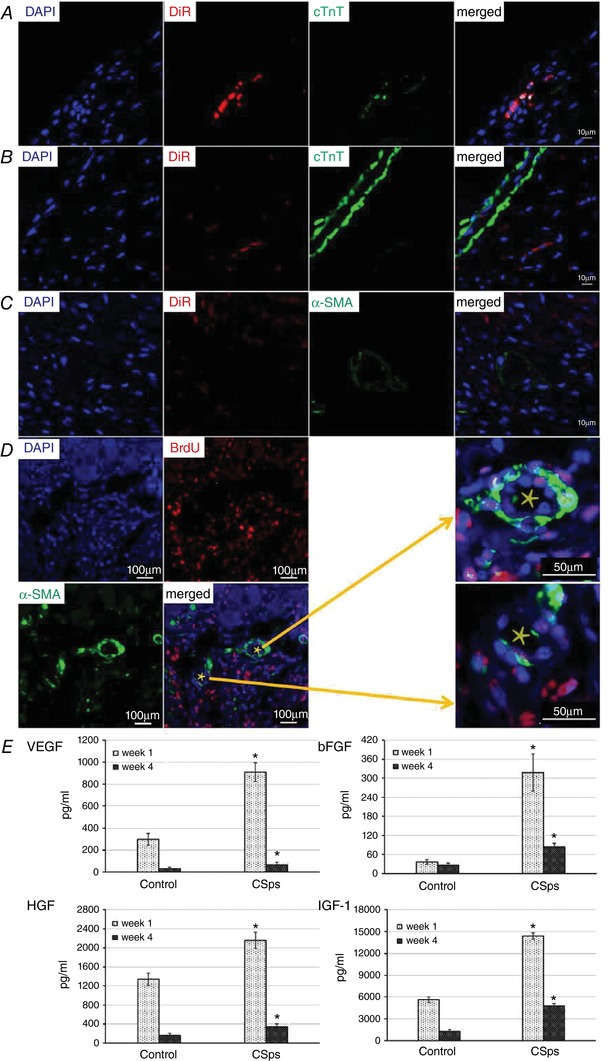
Differentiation of CSps into myocardial cells and the paracrine effect *A*, CSps differentiated into cardiac muscle cells directly after transplantation *in vivo*; *B*, CSps promoted the formation of cardiac muscle cells through the mechanism of the paracrine effect; *C* and *D*, transplantation of CSps *in vivo* promoted angiogenesis in infarcted myocardial tissue via a paracrine action; *C*, DiR‐labelled CSps did not overlap with α‐SMA‐labelled vessel; *D*, BrdU and α‐SMA staining showing the vessels were newly formed. Areas of blood vessels indicated by a star were enlarged as indicated by the arrows. *E*, contents of growth factors in fluid of the pericardial cavity. ^*^ *P* < 0.05 *versus* Control group.

To explore the mechanisms of action underlying the myocardial regenerative effect of CSp administration, we examined the differentiation of DiR‐labelled CSps into cardiomyocytes and development of blood vessels by direct immunofluorescence. Figure 7 *A* shows that transplanted DiR‐labelled CSps survived and infiltrated into the epicardium, and co‐staining DiR‐labelled cells with cTnT was also seen. The distribution of DiR only in DAPI‐positive areas excluded the possibility of non‐specific DiR dye leak into the acellular dead space. These data suggest that CSps can directly differentiate into myocardial cells after transplantation *in vivo*. Figure 7 *B* shows that CSps migrated into the infarcted myocardial tissue. The cTnT‐positive cells can be seen to be alongside the DiR‐labelled cells but rarely co‐stained with DiR. These results imply that CSps were able to promote the formation of cardiac muscle cells through a paracrine effect. In the present study, the mechanism by which CSps promote angiogenesis was also investigated. Figure 7 *C* shows α‐SMA‐positive cells around the DiR‐labelled CSps at the infarcted site, but there were no cells that expressed both α‐SMA and DiR. Figure 7 *D* shows additional BrdU staining, confirming that these vessels were newly formed. These results suggest that transplantation of CSps *in vivo* can promote angiogenesis in infarcted myocardial tissue via a paracrine action.

To ascertain the potential sources of paracrine mediators, the content of growth factors, VEGF, bFGF, HGF and IGF‐1, in pericardial cavity liquid was measured by ELISA 1 and 4 weeks after myocardial infarction. Figure 7 *D* shows that contents of VEGF, bFGF, HGF and IGF‐1 in pericardial effusion of the CSps group were significantly higher than those in the infarction only Control group 1 and 4 weeks after myocardial infarction. VEGF, bFGF, HGF and IGF‐1 contents in the CSps group were 3.06, 8.75, 1.61 and 2.54 times those in the Control group, respectively (*P* < 0.05) at 1 week after myocardial infarction (see also Table 1). At 4 weeks after myocardial infarction, VEGF, bFGF, HGF and IGF‐1 contents in the CSps group were 1.96, 3.12, 2.01 and 3.66 times those in the Control group, respectively (*P* < 0.05). These quantitative data indicate a large increase of the growth factors following CSp administration. These data therefore show that CSps intervention significantly enhanced the production of growth factors in the pericardial cavity, which could serve as paracrine mediators to stimulate proliferation and differentiation of cardiac muscle and blood vessels in the infarcted myocardium.

**Table 1:** Content of growth factors in fluid of the pericardial cavity

	CSps group		Control group	
	Week 1	Week 4	Week 1	Week 4
VEGF (pg mL^–1^)	909.0 ± 85.1**	65.3 ± 22.5**	297.4 ± 53.6	33.2 ± 12.2
bFGF (pg mL^–1^)	318.5 ± 58.9**	83.9 ± 11.2**	36.4 ± 6.9	26.9 ± 6.2
IGF‐1 pg mL^–1^)	14368.1 ± 423.4**	4786.1 ± 337.1**	5659.5 ± 382.5	1307.3 ± 247.8
HGF (pg mL^–1^)	2158.5 ± 167.6**	339.4 ± 64.2**	1338.6 ± 129.7	169.2 ± 36.2

^**^ *P* < 0.01 *versus* the control group at the same time point

**Table 2:** Two‐way ANOVA of cardiac function variables

		Sham	Control
**AWT**			
Week 0	Control	0.559	–
	CSps	0.807	0.732
Week 1	Control	0.002	–
	CSps	0.045	0.169
Week 2	Control	0.002	–
	CSps	0.034	0.204
Week 4	Control	<0.001	–
	CSps	0.002	0.075
**LVESD**			
Week 0	Control	0.172	–
	CSps	0.742	0.290
Week 1	Control	<0.001	–
	CSps	<0.001	0.004
Week 2	Control	<0.001	–
	CSps	<0.001	0.011
Week 4	Control	<0.001	–
	CSps	<0.001	0.002
**LVEF**			
Week 0	Control	0.556	–
	CSps	0.874	0.457
Week 1	Control	<0.001	–
	CSps	<0.001	<0.001
Week 2	Control	<0.001	–
	CSps	<0.001	<0.001
Week 4	Control	<0.001	–
	CSps	<0.001	<0.001

## Discussion

The results from the present study demonstrated that: (1) matrix hydrogel protected CSps against apoptosis and necrosis *in vitro* for up to 12 h; (2) 50% PFMI pretreatment enhanced the efficacy of CSps without affecting cell viability; (3) transplantation of matrix hydrogel‐packaged, 50% PFMI‐pretreated CSps into the pericardial cavity improved cardiac function and myocardial fibrosis following myocardial infarction, increased the survival of myocardial cells and promoted angiogenesis in the infarcted area: and (4) mechanistically, formation of cardiac muscle cells was mediated by direct differentiation of CSps as well as their paracrine action – angiogenesis was via a paracrine effect. To our knowledge, this is the first study that has administered pre‐optimized CSps via the pericardial cavity, demonstrated the effect of functional and morphological improvement following myocardial infarction and examined the underlying mechanisms.

### Cardiospheres as the therapeutic target stem cell

Previous studies have shown that CSps obtained from *in vitro* culture can simulate the growth microenvironment of cardiac stem cells in the body (Li *et al*. 2010), which helps the maintenance of cardiac stem cells. Our data show that the three cardiac stem cell markers c‐kit, sca‐1 and KDR were all expressed in the centre of CSps, which mimics the particular structure of cardiac stem cells in their growth microenvironment *in vivo*. These results further demonstrate that CSps can maintain the characteristics of cardiac stem cell. We noted that the locations of c‐kit, sca‐1 and KDR were different (Fig. 1) but the biological implication of this is currently not known. Our data also show that in P0–P2 generations, a greater number of CSps were formed, and the gene expression of growth factors VEGF, bFGF, HGF and IGF‐1 remained high. However, from P3 onwards, the number of CSps and the expression of growth factors started to decline. Therefore, the P2 generation of CSps was chosen as the target CSps for transplantation *in vivo*. This may be of relevance for CSps application in future studies.

### Matrix hydrogel packing for CSps *in vivo* transplantation

In clinical treatment, transport of stem cells from the laboratory to the operating theatre takes time. Ensuring the survival rate of stem cells during this period is an important factor in stem cell therapy (Chen *et al*. 2013). In previous studies, cold PBS was used to collect and store stem cells before transplantation *in vivo* (Tseliou *et al*. 2014). However, the survival state of the cells during this period has never been assessed. The present study assessed the viability of stem cells and found that CSps in PBS at 4°C began to become apoptotic after 1 h *in vitro*. The proportion of living cells continued to decline with time. After 6 and 12 h, living cells comprised only 86.35 ± 1.65 and 44.48 ± 1.45%, respectively. The present study assessed the protective effect of matrix hydrogel on CSps *in vitro*. The survival rate of CSps in the matrix hydrogel was significantly increased; the proportion of living CSps was 92.38 ± 1.43% after 12 h *in vitro* at 4°C (Fig. 2). Thus, matrix hydrogel can protect CSps against apoptosis or necrosis *in vitro* for up to 12 h. We therefore used CSps packaged by matrix hydrogel to be transplanted *in vivo*. These data suggest that matrix hydrogel can be a useful vehicle for maintaining viability during transportation of CSps stem cells.

### Enhancing CSps efficacy by PFMI pretreatment for *in vivo* transplantation

Following transplantation into the heart after myocardial infarction, the adverse living environment, especially hypoxia and inflammation, imposes a significant stress to CSps (Khan *et al*. 2010). To increase the survival and proliferation of stem cells after transplantation, many strategies have been explored previously. It was reported (Rosova *et al*. 2008; Yan *et al*. 2012) that hypoxic preconditioning could increase the survival and functioning of stem cells *in vitro* and *in vivo* after transplantation. In the present study, stem cells had to be transplanted into the pericardial cavity after myocardial infarction and survival in this post‐myocardial infarction PF was a significant challenge. A key question was how to improve the survival of CSps and promote their efficacy. We hypothesized that pretreatment of CSps with the PF from another subject undergoing myocardial infarction would enhance the ability of CSps to endure a subsequent myocardial infarction environment. CSps were pretreated with PFMI at different concentrations for 24 h *in vitro*. We found that 50% PFMI pretreatment significantly enhanced the biological activity of CSps, and also had no adverse effect on cell viability. Specifically, pretreatment with 50% PFMI enhanced mRNA expression of stem cell genes and differentiation of cardiac muscle cells, and promoted the expression of growth factor genes. These outcomes suggest that a high efficacy of CSps can be obtained by pretreatment with PFMI, a useful finding for stem cell therapy. The factors mediating the conditioning of CSps are unknown. Whether some ischaemia‐ and tissue‐necrosis‐stimulated growth factors play a role remains to be established. Furthermore, this PF conditioning effect is only observed on the CSps *in vitro*. Whether the observed enhancement of CSp activity directly contributes to myocardial regeneration requires further *in vivo* experiments by comparing the CSp implants.

### The pericardial cavity as the delivery route for CSps

The present study directly introduced CSps to the heart *in vivo* by intrapericardial administration. CSps have been shown to produce stem cells with better regenerative ability and low arrhythmogenicity, but have never been directly administrated via the pericardium to treat myocardial infarction. We have chosen the intrapericardial route for an important reason: as a solid cell cluster, CSps have the risk of entering the blood circulation and result in embolism if being delivered through intracoronary artery infusion (IC), interstitial retrograde coronary venous infusion (IRV) or intravenous infusion (IV) delivery routes; however, the pericardial cavity can retain the CSps, prevent microthrombolization and also limit the loss of stem cells into other tissue or the circulation. The results show that pericardial administration of CSps is safe. Of particular significance, we have also assessed the therapeutic potential of this manoeuvre for myocardial infarction with a 4‐week period of observation. The *in vivo* survival rate of DiR‐labelled CSps at different time points was assessed by a small animal *in vivo* imaging technique. The survival rate of CSps *in vivo* was 54.5 ± 7.5% at week 1, 31.0 ± 4.5% at week 2, and 16.8 ± 5.3% at week 4, much higher than the 2–11% retention rates for stem cells through other conventional routes of delivery (Hou *et al*. 2005). This demonstrates high retention and survival rate of CSps by intrapericardial delivery. We have also observed that all DiR‐labelled CSps were retained in heart tissue, with no leak to the left or right lung, in contrast to 25–50% of the delivered stem cells entering the lung (Hou *et al*. 2005). In addition, we have traced the outcome of CSps in cardiac tissue after transplantation and found that after 4 weeks of transplantation, significant DiR‐labelled cells survived in cardiac tissue sections. Importantly, these cells reached the epicardium, and also migrated into the infarcted myocardial tissues. These cells were thus able to contribute to myocardial regeneration. The use of the pericardial route to implant isolated stem cells has also been reported by two groups in previous studies (Saltzman *et al*. 2010; Blazquez *et al*. 2015, 2016) but no study has explored this route for application of CSps. Saltzman *et al*. (2010) tested endothelial progenitor cells for their ability to migrate into the area of infarct myocardium but did not examine myocardium‐derived stem cells. Blazquez *et al*. (2015, 2016) reported migration into the myocardial infarction zone by mesenchymal stem cells in one study and the safety of intrapericardial administration of isolated stem cells derived from CSps in another, although myocardium regeneration and ventricular function were not examined. Our findings provide the first evidence for the effective migration and incorporation into the infarcted myocardium by the CSps, which has distinct advantages over isolated stem cells from the CSp as discussed earlier

### Effect of CSp transplantation on myocardial infarction

The present study has shown that transplantation of these pre‐optimized and matrix hydrogel‐protected CSps into the pericardial cavity produced a significant therapeutic effect. (1) CSp transplantation improved cardiac function, as evidenced by significant improvements in LVESD, LVEF and survival rate. (2) This intervention ameliorated myocardial infarction‐induced myocardial fibrosis in the non‐infarcted area. (3) CSp transplantation increased surviving myocardial cells, as shown by the higher uptake rate of F‐FDG in the CSp group revealed by micro‐PET/CT. (4) This protocol allowed CSps stem cells to differentiate into myocardial cells, as shown by co‐staining cTnT and DiR (see below). (5) This treatment promoted angiogenesis in the infarcted area 4 weeks after myocardial infarction, as seen by increased α‐SMA‐positive staining in the CSps group. These data provide compelling evidence that pericardial CSps transplantation has high therapeutic potential for myocardial infarction. This is the first study that systematically examined myocardial remodelling and ventricular function in an experimental model of myocardial infarction by pericardial application of CSps and demonstrated consistent beneficial effects on all variables. A previous study under comparable experimental conditions using the SD rat model of myocardial infarction with a 4 week follow‐up showed that adipose‐derived stem cells improved LVEF by 20% while bone‐marrow‐derived stem cells had no effect; none of these cells reduced LVESD (Rasmussen *et al*. 2014). The 40 and 15% improvements on ejaculation fraction and end systolic diameter by CSps in our study demonstrate an advantage over other stem cell therapies in this earlier study.

### Direct differentiation and paracrine action as mechanisms of regeneration

The present study examined direct differentiation of CSps into cardiomyocytes *in vivo* and as well as their indirect effect on cardiac cell proliferation and angiogenesis by DiR labelling in combination with immunofluorescence. We found that some transplanted DiR‐labelled CSps were also cTnT‐positive. This demonstrates that CSps can differentiate into cardiac muscle cells directly after transplantation *in vivo*. There was also significant accumulation of cTnT‐positive cells around DiR cells but they were not co‐stained with DiR, consistent with CSps exerting an indirect trophic effect on adjacent cardiac cells by paracrine action. However, no cells co‐expressed α‐SMA and DiR. This suggests that CSps do not differentiate into blood vessels directly. Instead, a large number of α‐SMA‐positive cells, which were not co‐stained with DiR, surrounded DiR‐labelled cells. These data suggest that the increase in blood vessels was mediated by a mechanism of paracrine effect. Both direct differentiation and paracrine effect are important underlying mechanisms for improved cardiac function and remodelling by intrapericardial CSps. We also investigated potential mediators which could augment the efficacy CSps themselves in the pericardial cavity and generate a paracrine effect in the infarcted myocardium. Direct assessment of the content of growth factors showed that VEGF, bFGF, HGF and IGF‐1 in pericardial effusion from the CSps group were many fold higher than those from the Control group at both early and late time points after myocardial infarction. These findings demonstrate that CSps transplantation can increase these growth factors in the pericardial space, which could serve as paracrine mediators to promote myocardial cell growth and angiogenesis in the infarcted area. The ability of these growth factors to promote myocardial regeneration and angiogenesis has been described by previous studies (Segers & Lee, 2010; Marsano *et al*. 2013; Chen *et al*. 2015). More specific experiments are required to identify the mediators responsible for the paracrine stimulation of myocardial regeneration by pericardial CSps implantation.

## Conclusions

Pretreatment of CSps with PFMI can enhance mRNA expression of stem cell genes and differentiation of cardiac muscle cells, and promote expression of growth factor genes; this suggests that high biological activity of CSps can be obtained by PFMI treatment. Matrix hydrogel can protect CSps against apoptosis and necrosis *in vitro* for up to 12 h. Transplantation of PFMI‐preconditioned, matrix hydrogel‐protected CSps into the pericardial cavity can improve cardiac function, reduce myocardial fibrosis, increase the survival of myocardial cells and promote angiogenesis in the infarcted area. Mechanistically, CSps can be directly differentiated into cardiac myocytes *in vivo*, and promote regeneration of cardiac myocytes and blood vessels in the infarcted area through a paracrine mechanism. This is the first application of modulatable CSps implantation into the pericardial cavity to treat myocardial infarction in an experimental model with a positive outcome.

## Additional information

### Competing interests

The authors declare no conflicts of interest.

### Author contributions

Jianhua Zhang, Zheng Wu and Jingfeng Wang: conception and design, collection, and/or assembly of data, data analysis and interpretation, manuscript writing, final approval of manuscript; Zepei Fan, Zixi Qin and Yingwei Wang: cell culture and detection of data in the *in vitro* experiment; Jiayuan Chen and Maoxiong Wu: effective support in animal experiments; Yangxin Chen: conception, design, and manuscript writing; Changhao Wu: conception, design, interpretation, critical analysis and manuscript writing, final approval of the manuscript.

### Funding

This work was supported by the National Natural Science Foundation of China (91439125), the Special Financial Grant from the China Postdoctoral Science Foundation (2015T80937), National High Technology Research and Development Program for Young Scientists of China (2014AA020534), Science and Technology Planning Project of Guangdong Province (2013B010404030), Research Project of Scientific Research Cultivation and Innovation Fund of Jinan University (21617488), and Project of Innovative and Entrepreneurial Training Program for College Students in Jinan University (national level: 201710559035, and school level: CX17024). CW gratefully acknowledges support from Biotechnology and Biological Sciences Research Council (BBSRC) (BB/I025379/1, BB/P004695/1) and National Institute of Aging (NIA, 1R01AG049321‐01A1).
